# Tracing the reconfiguration of performance ecology in Quanfeng Huadeng: an ethnographic study of heritage governance, agency, and participation

**DOI:** 10.3389/fpsyg.2026.1866204

**Published:** 2026-08-11

**Authors:** Lu Liang, Zhou Jun, Shi Jun

**Affiliations:** 1 Faculty of Music, Xinyu University, Xinyu, Jiangxi, China; 2 Faculty of Music and Performing Arts, Sultan Idris Education University, Tanjung Malim, Perak, Malaysia

**Keywords:** agency, China, ethnography, folk opera, heritage governance, intangible cultural heritage, participation, performance ecology

## Abstract

**Introduction:**

Quanfeng Huadeng, a small-scale folk opera from Xiushui County, Jiangxi Province, has historically been embedded in rural festival life through singing, dance, lantern display, comic narration, local dialect, and close audience–performer interaction. Since its inscription on China's National Intangible Cultural Heritage list in 2006, Huadeng has increasingly moved from flexible village-based performance contexts toward curated, staged, and institutionally organized forms of cultural presentation. This study examines how this transition has reconfigured its performance ecology, understood as the interdependent system of temporal rhythm, spatial arrangement, performer composition, aesthetic orientation, and participatory relations through which performance becomes socially meaningful.

**Methods:**

The study drew on phased ethnographic engagement in Quanfeng Township between 2021 and 2026, with principal evidence obtained through formal doctoral fieldwork conducted in 2025. Data included participant observation, semi-structured interviews with 21 core participants, approximately 30 brief audience interviews, group conversations and immediate audience feedback records, documentary materials, visual evidence, and audio-visual records. Analysis involved iterative thematic coding and triangulation across interviews, field notes, policy and archival materials, photographs, and performance recordings.

**Results:**

Findings were organized around four interrelated dimensions: rhythm, spatial form, performer composition, and aesthetic orientation. Heritage governance brought visibility, resources, and new transmission opportunities, but also introduced fixed performance durations, staged spatial separation, managed performer organization, and visually oriented aesthetic criteria. These transformations reorganized the conditions through which improvisation, community knowledge, audience interaction, situated engagement, and practitioner agency were enacted.

**Discussion:**

By bringing structuration theory into dialogue with cultural and ecological psychology, the study conceptualizes performance ecology transformation as a case-based analytical tool for examining how institutional rules and resources are negotiated within lived performance practice. Sustainable safeguarding should protect not only repertoire and visible form, but also the social, spatial, temporal, and participatory conditions that allow living heritage to remain locally meaningful.

## 1 Introduction

Quanfeng Huadeng (全丰花灯) is a small-scale folk opera originating from Quanfeng Township and surrounding villages in Xiushui County, Jiangxi Province. Integrating singing, dance, lantern display, comic narration, local dialect, and situational humor, it has historically functioned as a flexible, community-rooted performance tradition embedded in the rhythms of rural festival life (Zhou, 2020, 2021). Traditionally performed during the Spring Festival and Lunar New Year period, Huadeng was organized informally by villagers and staged in open social spaces such as *tangqian* (堂前), village entrances, courtyards, and public squares. Audiences were usually composed of neighbors, relatives, and long-term acquaintances. In these contexts, performance was not only a form of entertainment but also a social occasion through which local identity, interpersonal ties, and shared community memory were enacted and renewed (Zhou, 2020, 2021).

The distinctiveness of Huadeng lies in the way performance form is tied to local social knowledge. Its repertoire draws on moral tales, marriage customs, rural life, agricultural experience, and everyday interpersonal relationships. Pieces such as *Persuading My Husband* (*Quanfu* 劝夫), *Selling Groceries* (*Maizahuo* 卖杂货), and 10 *Months of Pregnancy* (*Shiyue Huaitai* 十月怀胎) were not performed as fixed theatrical texts alone, but were selected and adapted according to the host family, festive occasion, or social situation. Its plain language, comic narration, and improvised monologs (*jiangbai* 讲白) enabled audiences to recognize familiar people, problems, and values within the performance (Zhang, 2021). In this sense, Huadeng can be understood as a lived performance ecology: a relational system in which temporal rhythm, spatial proximity, performer knowledge, audience response, humor, and local meaning are mutually connected.

This ecological quality is important because Huadeng's earlier village-based form depended on conditions that were both artistic and social. Performers adjusted rhythm, song choice, spoken lines, and comic timing in response to audience mood and household context. Audiences were not passive spectators but co-present participants who could laugh, correct lyrics, respond verbally, request songs, or evaluate performance through immediate feedback. Such participation is not simply an audience reaction after the performance; it forms part of the performance structure itself (White, 2013). From this perspective, Huadeng may be understood as a situated cultural practice in which identity, memory, musical meaning, and social relation are enacted through embodied participation (MacDonald and Saarikallio, 2022; Miller and Chauhan, 2012; Valsiner, 2013; Wan and Chew, 2013). However, in this article, psychological engagement is approached interpretively through ethnographic evidence rather than treated as a directly measured psychological variable.

Since the 1990s, the continuity of community-based Huadeng practice has been affected by urbanization, rural demographic change, aging performer groups, and shifting media consumption, a pattern also observed in other rural folk performance traditions in China (Xu, 2014). A senior male performer reflected that “over the past three decades, the number of active Huadeng performers has steadily diminished, and fewer young people are willing to learn” (personal interview, February 9, 2021). These pressures weakened the everyday social base that previously sustained Huadeng as an informal festive practice. At the same time, they created conditions under which formal heritage intervention became increasingly important for ensuring visibility, documentation, and institutional support.

A major turning point occurred in 2006, when Quanfeng Huadeng was inscribed on China's National Intangible Cultural Heritage list. This recognition brought new resources, public visibility, training opportunities, and official legitimacy. Yet it also repositioned Huadeng within a broader heritage governance framework shaped by cultural policy, public display, administrative evaluation, tourism promotion, and the designation of representative inheritors (Dutournier and Padovani, 2021; Li et al., 2025; Zhu, 2026). In China, ICH governance has increasingly formalized local practices through policy discourse, institutional arrangements, resource mobilization, and regulatory mechanisms, often transforming community-led practices into more formalized and spatially organized cultural systems (Li et al., 2025). Similar processes have also been observed in other Chinese performing traditions, where institutionalized troupe systems support continuity while reshaping performer roles, cultural authority, and modes of reproduction (Yang and Wu, 2026). In this process, Huadeng increasingly moved from flexible village-based performance toward curated cultural showcases, staged programmes, publicity performances, and tourism-oriented presentations.

This problem connects with wider debates in intangible cultural heritage studies. Heritage designation is rarely a neutral act of preservation. It often involves selecting, standardizing, documenting, and presenting living practices in ways that make them legible to institutions, tourists, policy agencies, and wider publics (Kirshenblatt-Gimblett, 2004; Li et al., 2025; Norton and Matsumoto, 2018; Zhu, 2012). In the Chinese context, this process is also shaped by the tension between preservation and development. Heritage practices are expected to remain culturally meaningful while also serving public education, local branding, tourism development, and regional cultural economy. Studies of Chinese heritage markets, rural heritage development, and tourism-oriented performance show that institutional and market forces can expand visibility and value, but may also reshape local meanings, participation, and cultural authority (Chen et al., 2021; Maags, 2021; Milan, 2023; Su, 2023). The issue is therefore not simply whether heritage governance preserves or damages tradition, but how it reorganizes the conditions under which performance is produced, evaluated, and experienced.

Existing scholarship on Quanfeng Huadeng has contributed valuable knowledge on its artistic characteristics, repertoire, cultural significance, and potential for regional music education and institutional transmission (Zhang, 2021; Zhou, 2020, 2021). However, less attention has been given to how Huadeng's live performance ecology changes when it moves from village-based social contexts into heritage-governed and institutionally staged settings. Broader studies of small-scale opera and regional performance traditions, such as Jie Dang (街檔) Cantonese opera in Hong Kong, Pu Kang folk opera, and Shanghai Yue Opera (YueJu), demonstrate the scholarly value of examining performance traditions within their specific socio-cultural and institutional settings (Haili, 2012; Wang, 2020; Wong, 2016). Yet Quanfeng Huadeng remains underexplored as an ethnographic case for understanding how heritage governance reshapes the interaction between structure, agency, participation, and local performance knowledge.

This study addresses that gap by examining the reconfiguration of Quanfeng Huadeng's performance ecology under conditions of heritage governance. Performance ecology is used here as an analytical concept referring to the interdependent system of temporal rhythm, spatial arrangement, performer composition, aesthetic orientation, audience relation, and local knowledge through which performance becomes socially meaningful. Rather than treating rhythm, space, personnel, and aesthetics as separate categories, the study examines how these dimensions work together as a system. A change in performance time, for example, may reduce opportunities for improvised dialogue; a change in performance space may alter audience visibility and body orientation; a change in performer recruitment may affect dialect humor and shared cultural memory; and a change in aesthetic criteria may shift participation from reciprocal interaction toward spectatorial appreciation. The study is guided by three research questions:

1. How has heritage governance reshaped the temporal, spatial, organizational, and aesthetic conditions of Quanfeng Huadeng performance?
2. How do performers, spectators, apprentices, and cultural officials interpret and negotiate these changes?
3. How do these transformations affect the conditions through which participation, improvisation, agency, and engagement are enacted in performance practice?

By addressing these questions, the article contributes to intangible cultural heritage scholarship in three ways. First, it offers a sustained ethnographic account of Quanfeng Huadeng, a tradition that has received limited attention in English-language scholarship. Second, it develops the concept of performance ecology transformation to explain how heritage governance affects not only visible cultural form but also the relational conditions that sustain live performance. Third, by bringing structuration theory into dialogue with cultural and ecological psychology, the study analyses heritage transformation as an ongoing negotiation between institutional rules and resources, practitioner agency, and the situated conditions of participation. The argument is therefore not that heritage governance simply causes loss, but that it creates new possibilities while simultaneously reorganizing the social, spatial, temporal, and participatory conditions through which Huadeng remains meaningful as living heritage.

### 1.1 Theoretical context and analytical framework

The transformation of Quanfeng Huadeng following its inscription on the National Intangible Cultural Heritage list reflects a broader pattern in which community-based cultural practices are reorganized through heritage governance, public display, and institutional safeguarding. Heritage designation is rarely a neutral act of preservation. Rather, it often entails processes of selection, documentation, standardization, framing, and re-presentation through which living traditions are made legible to cultural institutions, policy agencies, tourists, and wider publics. Kirshenblatt-Gimblett (2004) conceptualizes this process as metacultural production, whereby heritage practices are reflexively reconstructed to meet representational, institutional, and audience expectations. In the context of music and performance, such processes may preserve visibility and continuity while also altering the social conditions through which performance is learned, enacted, and valued (Li et al., 2025; Norton and Matsumoto, 2018; Zhu, 2012, 2026).

In the Chinese context, intangible cultural heritage governance has expanded through policy frameworks that prioritize documentation, representative inheritor systems, public promotion, educational transmission, and local cultural branding (Dutournier and Padovani, 2021; Li et al., 2025). These mechanisms provide important resources for local traditions, including recognition, funding, rehearsal opportunities, training platforms, and new audiences. At the same time, they may also produce new forms of administrative authority over what counts as legitimate heritage practice. Recent studies of Chinese intangible cultural heritage show that heritage governance often operates at the intersection of preservation, tourism, rural development, market value, and cultural policy (Chen et al., 2021; Maags, 2021; Su, 2023; Zhu, 2026). This tension between safeguarding and developmental use is central to the present study because Quanfeng Huadeng has not simply been preserved as an inherited form; it has increasingly been repositioned within staged performances, publicity events, tourism-oriented presentations, and institutional training contexts.

Within performance traditions, metacultural production often appears through the formalization of repertoire, the professionalization of performers, and the restructuring of performance settings for external audiences. These processes can expand the reach and public value of a tradition, but they may also change the conditions of audience participation, performer agency, and local knowledge. For example, studies of heritage markets and rural cultural development in China show how heritage practices may be transformed into cultural resources for economic development and public display (Chen et al., 2021; Maags, 2021). Similarly, research on institutionalized performing troupes demonstrates that formal organizational systems can support continuity while also reshaping cultural reproduction, performer roles, and authority structures (Yang and Wu, 2026). These debates are especially relevant to Huadeng, where village-based festive performance increasingly coexists with institutional showcases, policy-themed performances, and staged programmes.

To analyse this process, the study uses the concept of performance ecology transformation. Performance ecology refers here to the interdependent system of temporal rhythm, spatial arrangement, performer composition, aesthetic orientation, audience relation, local knowledge, and participatory practice through which performance becomes socially meaningful. The concept is not used as a synonym for performance change. Rather, it identifies the relational conditions that allow a living performance tradition to function. In Huadeng, a change in one ecological dimension often affects the others. Fixed performance durations may reduce opportunities for improvised dialogue; staged spatial arrangements may alter visibility, body orientation, and audience response; the introduction of external or semi-professional performers may affect local dialect humor and shared social knowledge; and aesthetic formalization may shift attention from participatory wit toward visual polish and representational clarity.

This ecological framing also draws on cultural and ecological psychology, but in a focused and cautious way. Cultural psychology emphasizes that meaning, identity, and social relations are mediated through culturally organized practices and symbolic systems (Miller and Chauhan, 2012; Valsiner, 2013; Wan and Chew, 2013). Ecological approaches further suggest that environments are not neutral containers for action; they shape what kinds of perception, movement, interaction, and participation become possible (De Young, 2013; Kals et al., 2025). In Huadeng, performance spaces such as village halls, courtyards, household interiors, cultural centers, and stage venues offer different affordances for audience proximity, eye contact, verbal response, and performer adaptation. Thus, changes in spatial setting are also changes in the conditions of participation.

The psychological dimension of this study is therefore treated as an interpretive dimension of ethnographic analysis, not as a directly measured psychological outcome. Engagement is understood as involving behavioral, emotional, and cognitive dimensions (Fredricks, 2015), but the study does not claim to measure these dimensions psychometrically. Instead, it examines how performance conditions make particular forms of engagement more or less available. Behavioral engagement is examined through visible participation, call-and-response, audience movement, performer adaptation, and rehearsal involvement. Emotional engagement is examined through participants' accounts of attachment, enjoyment, pride, discomfort, nostalgia, and alienation. Cognitive engagement is examined through attention, interpretation, improvisational decision-making, and shared understanding of lyrics, humor, and local references. This approach allows the article to discuss psychological engagement without overstating the evidentiary basis of the ethnographic data.

Improvisation provides a further analytical link between performance ecology and engagement. In Huadeng, improvisation is not merely individual spontaneity. It depends on shared repertoire, local dialect, knowledge of households, audience familiarity, comic timing, and the capacity to adjust performance in real time. Studies of improvisation and collaborative creativity similarly emphasize that creative performance emerges through interaction, situated responsiveness, and social coordination rather than isolated invention (Kenny and Gellrich, 2002; MacDonald and Saarikallio, 2022; Monzo et al., 2025; Sawyer, 2003). From this perspective, the standardization and scripting associated with heritage governance are not only aesthetic changes. They may also alter the conditions that enable situated creativity, performer agency, and audience-responsive meaning-making.

Giddens (1984) structuration theory provides the main explanatory lens for analyzing how these transformations occur. Structuration theory is useful because it avoids treating heritage governance as a one-way imposition from institutions onto passive local practitioners. Its core concept, the duality of structure, suggests that social structures are both the medium and the outcome of practice. Structures consist of rules and resources that constrain and enable action, while actors reproduce, negotiate, and sometimes modify these structures through everyday practice. In the case of Huadeng, the relevant rules include fixed programme duration, approved performance themes, rehearsal requirements, inheritor designation, evaluation criteria, and expectations of stage suitability. The relevant resources include funding, training opportunities, official recognition, costumes, venues, publicity platforms, and access to wider audiences.

This framework allows the study to examine how practitioners mobilize or negotiate institutional rules and resources in specific performance scenarios. For example, performers may shorten traditional narratives to fit an 8-min stage slot, modify *jiangbai* to align with policy themes, use institutional funding to support costumes and training, or preserve more flexible forms of interaction during village-based performances. Such practices show that local actors are neither simply resisting nor simply accepting heritage governance. Rather, they engage in situated negotiations. Recent research on Chinese heritage practice similarly shows how practitioners negotiate between policy demands, public platforms, and local cultural logics (Yi, 2023), while institutionalized troupe systems demonstrate how cultural reproduction is reshaped through formal organizational structures (Yang and Wu, 2026). In this sense, structuration theory helps explain how Huadeng's transformation emerges through the ongoing interaction between institutional arrangements and practitioner agency.

Taken together, these perspectives provide the analytical framework for the article. Heritage governance is understood as a system of rules and resources. Performance ecology transformation is understood as the reorganization of the relational conditions through which performance is produced, experienced, and evaluated. Psychological engagement is understood as an interpretive dimension of participation, inferred from ethnographic evidence rather than directly measured. This framework allows the study to examine Quanfeng Huadeng not as a static heritage object, but as a living performance system in which rhythm, space, performer composition, aesthetics, agency, and participation are continuously negotiated.

## 2 Methodology

This study adopted a qualitative ethnographic design to examine how heritage governance has reconfigured the performance ecology of Quanfeng Huadeng. The focus was not only on changes in performance form, but also on how performers, spectators, cultural officials, organizers, and local audiences experienced and negotiated changes in rhythm, spatial arrangement, performer composition, aesthetic orientation, and participation. Ethnography was appropriate because these transformations are embedded in lived practice, local relationships, performance settings, oral memory, and institutional arrangements rather than in variables that can be separated from context (Denzin and Lincoln, 2011; Pink, 2015). The study combined participant observation, semi-structured interviews, brief audience interviews, informal conversations, documentary materials, and visual ethnographic evidence to examine Huadeng as a situated cultural practice.

### 2.1 Research design

The research was designed as a long-term, site-based ethnographic inquiry conducted in natural performance and rehearsal settings. Rather than reconstructing Huadeng as a staged research event, the study followed the tradition across village-based festive performances, household performances, processional performances, rehearsals, institutional showcases, cultural publicity events, and tourism-oriented presentations. This enabled comparison across community-embedded and institutionally organized contexts.

The researcher moved between observer and participant-observer roles depending on the setting. During public performances, the researcher primarily adopted an audience perspective, documenting performer–audience interaction, spatial organization, performance duration, body orientation, audience proximity, repertoire selection, and moments of verbal or non-verbal response. In rehearsal, backstage, and community settings, the researcher engaged more closely with performers and organizers through informal conversations, observation of rehearsal decisions, and limited practical assistance where appropriate. This shifting positionality enabled both contextual intimacy and analytical distance (Pink, 2015; Tanu and Dales, 2016).

Reflexivity was embedded throughout the research design. The researcher maintained field notes and reflexive memos to document how access, rapport, linguistic competence, institutional affiliation, and perceived insider–outsider position shaped interactions with performers, cultural officials, organizers, and spectators. These memos informed subsequent data collection and interpretation, particularly where participants discussed sensitive issues such as authenticity, government expectations, performer substitution, changing audience reception, and the distinction between village-based and official performance contexts (Folkes, 2023; McCorkel and Myers, 2003).

### 2.2 Fieldwork site and corpus

The study was informed by phased field engagement with Quanfeng Huadeng between 2021 and 2026. Materials collected between 2021 and 2024 were treated as preliminary field engagement that helped establish historical trajectories, identify key participants, understand local performance contexts, and refine the research questions. This phased approach is consistent with ethnographic research, where prolonged contextual familiarity and iterative engagement are important for understanding cultural practice *in situ* (Denzin and Lincoln, 2011; Pink, 2015).

Second, formal doctoral fieldwork commenced on 1 March 2025 and continued for 6 months in Quanfeng Village, Quanfeng Township, Xiushui County, Jiangxi Province. This period provided the principal basis for systematic interviews, verification of earlier materials, and detailed analysis in relation to the research questions. Third, supplementary fieldwork in January 2026 was used to observe the Third Quanfeng Huadeng Arts Festival and to verify selected findings from the formal doctoral fieldwork. The supplementary materials did not alter the principal fieldwork period but strengthened the interpretation of changing performance practices within formal staged contexts.

The core interview dataset consisted of 21 participants, including Huadeng performers, senior representative inheritors, troupe leaders, local cultural officials, village audience members, and members of the Quanfeng Huadeng Research Association. In addition, the study drew on approximately 30 instances of brief audience interviews, group conversations, and immediate audience feedback recorded during Chinese New Year village performances and formal staged performances. These brief audience responses were not treated as independent core interview cases, but were used to understand audience reception, listening expectations, and immediate evaluations of performance. Across the fieldwork, the study generated approximately 60 h of audio recordings, 10 h of video recordings, field notes, monolog transcriptions, repertoire documentation, local documentary sources, and materials relating to ICH nomination and representative inheritors.

### 2.3 Participants and recruitment

Participants were recruited through purposive and snowball sampling. Purposive sampling was used to identify information-rich participants with direct experience of Huadeng performance, transmission, organization, administration, or spectatorship, while snowball sampling helped identify additional participants through local networks and practitioner recommendations (Campbell et al., 2020; Noy, 2008; Palinkas et al., 2015). The aim was not statistical representativeness, but analytical depth across stakeholder positions.

The 21 core participants included performers, senior representative inheritors, troupe leaders, local cultural officials, village audience members, and members of the Quanfeng Huadeng Research Association. Because individuals involved in Huadeng often hold multiple roles, participant categories were not treated as mutually exclusive statistical groups. For example, one individual may simultaneously be a representative inheritor, performer, troupe organizer, or member of the local research association. The participant categories were therefore used to show the range of perspectives contributing to the study rather than to create a fixed demographic sample.

To maintain transparency while protecting confidentiality, the manuscript reports participants by role category, gender, age group or year of birth, and interview context where appropriate. Some participants are identified by real name only where they gave consent and where the information relates to public cultural roles or non-sensitive factual matters. For quotations involving sensitive evaluations, institutional critique, or non-public opinions, pseudonyms or participant codes are used.

**Figure 1:**
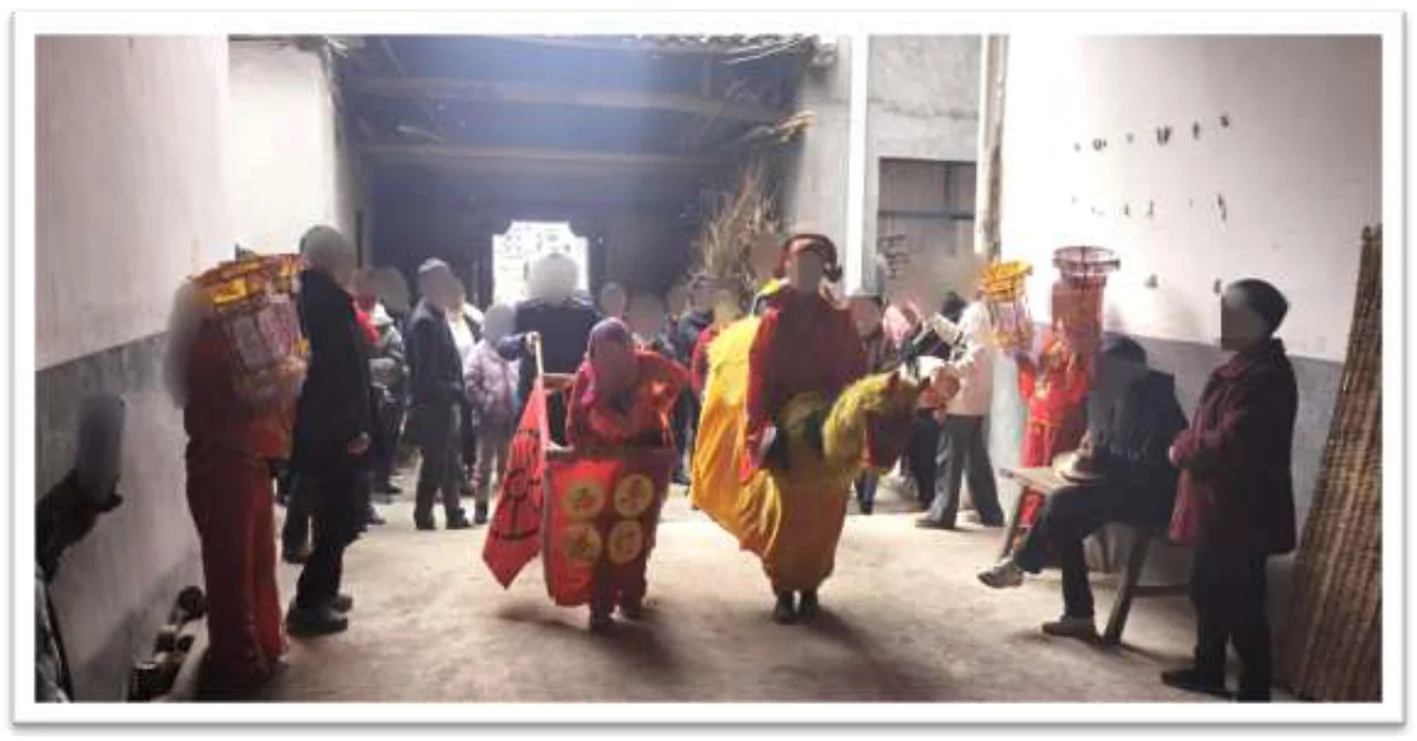
During the Chinese New Year in 2025, a Huadeng team performs in the main hall of a traditional village residence. Source: Authors.

**Figure 2:**
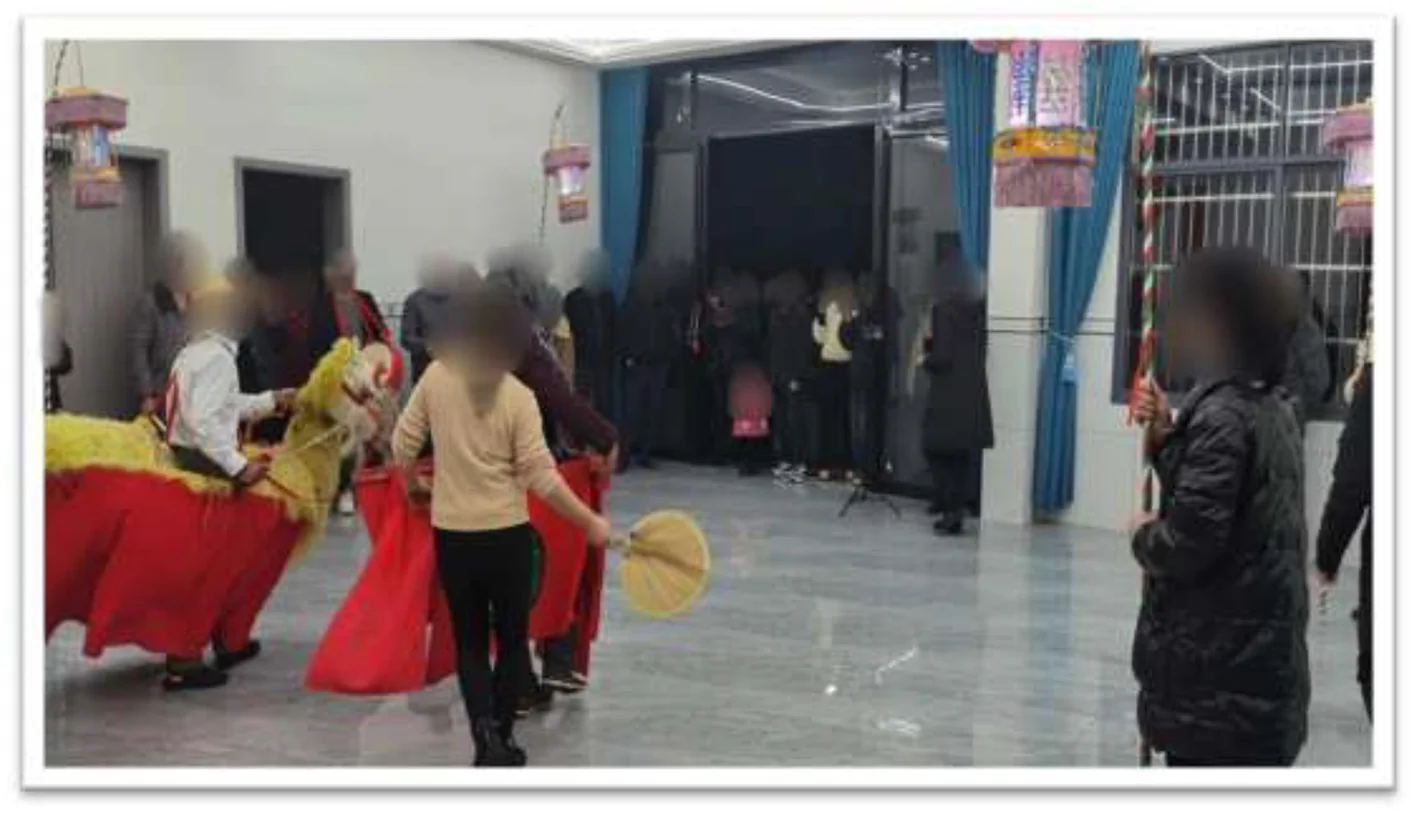
During the 2024 Chinese New Year, the Longquanduan Huadeng team performs in the main hall of a newly constructed village house. Source: Authors.

**Figure 3:**
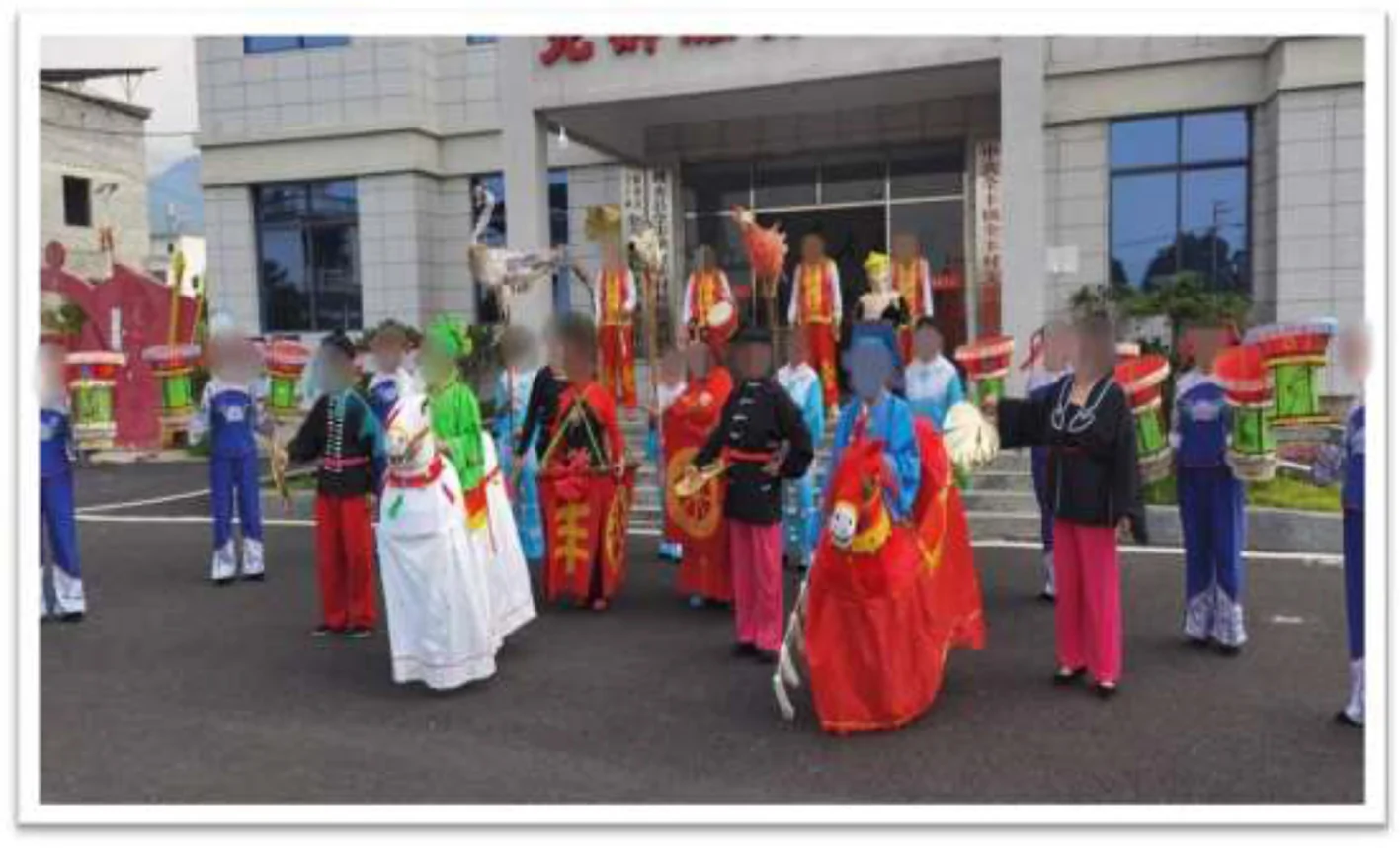
Final rehearsal/performance preparation by the Huadeng team of the Quanfeng Village Cultural Center for a legal publicity performance organized by the county judicial department. Source: Authors.

To protect participant confidentiality while maintaining a transparent evidence trail, core interview participants are identified in the Results using participant codes P1–P21. Brief audience interviews, group conversations, and immediate audience feedback records are identified as audience feedback records, coded A1–A30 where relevant. Field notes are identified as FN, video records as VR, audio records as AR, and photographic materials as Figures 1–3. This coding system allows the article to show how findings were developed across multiple data sources without disclosing unnecessary personal information. Where participants held public cultural roles, identifying details were still minimized unless disclosure was necessary, non-sensitive, and consented to.

### 2.4 Interview, language and translation

Semi-structured interviews explored participants' experiences of Huadeng before and after heritage designation, perceptions of institutional intervention, changes in performance rhythm and space, performer recruitment, aesthetic expectations, audience relations, and local strategies of adaptation. Interview questions were used flexibly to allow participants to foreground locally meaningful concerns rather than merely responding to researcher-imposed categories.

Interviews were conducted in Mandarin, local dialect, or both, depending on the participant and the performance context. Mandarin was used particularly with non-local audience members, while local dialect was important when interviewing performers and village participants because Huadeng relies heavily on dialect-based humor, monolog, rhythm, and context-specific meaning. Interview materials were audio-recorded with consent where appropriate, and field notes were maintained for informal conversations and immediate audience responses.

Translation was treated as part of the analytical process rather than as a straightforward linguistic transfer. Materials moved from Quanfeng dialect to Standard Mandarin and then into English for manuscript preparation. Culturally specific keywords, role types, repertoire titles, and performance terms were retained in Chinese or pinyin where necessary, with explanatory glosses at first mention. This was important because the meaning of jiangbai monolog, comic timing, rhyme, and dialect humor could not always be captured through literal translation alone. The translated English manuscript was reviewed for clarity and naturalness, while the interpretation of dialect-based terms was checked against the performance context, interview recordings, and field notes.

### 2.5 Participant observation, documentary materials and visual data

Participant observation was conducted across multiple performance contexts, including village-based festive performances, household performances, processional performances, rehearsals, officially organized heritage showcases, policy-themed publicity performances, and tourism-oriented stage productions. Observing the same tradition across contrasting contexts enabled analytical comparison between community-embedded and institutionally staged formats.

Field notes documented performance duration, repertoire selection, staging, performer formation, audience location, costume, lighting, sound, use of subtitles, performer–audience interaction, moments of humor, audience correction, applause, silence, discomfort, and backstage negotiation. For example, village-based contexts showed closer performer–audience proximity and opportunities for spontaneous comment, while municipal, publicity, or tourism performances often involved compressed duration, formal costumes, fixed audience areas, and limited interaction.

Documentary and audiovisual materials included scripts, photographs, local promotional materials, internal documents, ICH nomination materials, representative inheritor records, rehearsal records, video recordings, and audio recordings. These materials were treated not as neutral evidence, but as cultural and institutional artifacts that reflect processes of framing, selection, documentation, and representation within heritage governance (Kirshenblatt-Gimblett, 2004; Zhu, 2012).

Visual materials were used as visual ethnographic evidence rather than decorative illustration. Across the wider fieldwork, the researcher collected approximately 1,000 photographs and more than 80 video and audio recordings. The three photographs included in the Results were selected because they represent three contrasting spatial configurations: village-based co-present performance, transitional household performance, and institutionally organized staged rehearsal or performance. The images were analyzed in relation to visual accessibility, performer–audience distance, spatial boundaries, body orientation, direction of attention, and opportunities for interaction. This approach is consistent with visual ethnographic methods that treat images as part of field-based interpretation rather than merely as records of what was seen (Pauwels, 2009; Pink, 2015).

The three photographs presented in the Results were selected from the wider visual corpus because each represents a distinct spatial configuration: village-based co-present performance, transitional household performance, and institutionally organized staged or rehearsal-based performance. They were analyzed in relation to performer–audience distance, spatial boundaries, body orientation, direction of attention, and opportunities for interaction. Visual materials were therefore treated as part of the analytical evidence rather than as decorative illustrations.

### 2.6 Data analysis and triangulation

Data analysis followed an iterative thematic approach. Interview transcripts, field notes, performance records, documentary materials, and visual materials were read and reviewed repeatedly to identify patterns in how Huadeng transformation was experienced, narrated, and observed across different contexts (Braun and Clarke, 2006). The first stage involved open reading and initial coding. Initial codes included Chinese New Year processional performances, government-organized performances, adaptation of monolog, audience feedback, gong-and-drum coordination, repertoire selection, ICH training, stage compression, performers' memories, and interaction with host families.

The second stage involved thematic categorization. Initial codes were grouped into broader analytical categories, including historical memory, institutional mechanisms, performance settings, sound and rhythmic organization, performer strategies, and audience reception. These categories were then refined in relation to the article's four analytical dimensions: rhythm, spatial form, performer composition, and aesthetic orientation. The third stage involved cross-material comparison. Narratives provided by different participants, observations conducted in different years, formal and informal performance contexts, interview data, documentary materials, and audio-visual records were compared in order to avoid drawing conclusions from a single source of evidence.

In the Results, each analytical theme is supported through cross-material comparison rather than by a single interview source. For example, the theme of temporal compression was developed by comparing performer interviews, field notes, and video records from village-based and staged performances. The theme of spatial transformation was developed through comparison of photographs, observation notes, and audience feedback. The theme of performer composition was developed through performer interviews, official or organizer accounts, rehearsal observations, and documentary materials. The theme of aesthetic orientation was developed through performer accounts, audience feedback, staged-performance observations, and audio-visual records. This procedure was used to make the analytical pathway from data to theme explicit.

The final stage involved theoretical interpretation. The analytical themes were examined in relation to heritage governance, structuration theory, performance ecology, and audience interpretation. For example, shortened performance duration was interpreted not merely as a technical adjustment, but as a sign of institutional time discipline and altered conditions for improvisation. Similarly, changes in stage arrangement were interpreted in relation to performer–audience distance, body orientation, and the affordances available for reciprocal interaction.

Triangulation was used to strengthen interpretive credibility by comparing interviews, observations, documents, and visual materials. The purpose was not to force all sources into agreement, but to examine convergence, complementarity, and tension across different forms of evidence (Denzin and Lincoln, 2011). For example, performers' accounts of shortened performance time were compared with observations of staged performances; narratives about weakened dialect humor were compared with observations of modified jiangbai and the use of subtitles; and claims about performer professionalization were compared with rehearsal observations and institutional materials. Local cultural department documents, ICH nomination materials, and representative inheritor records were cross-checked against performers' oral accounts, field observations, local documentary sources, and audio-visual materials. At the same time, these documents were analyzed as forms of heritage discourse and institutional representation in their own right.

### 2.7 Reflexive positionality

Reflexivity was treated as a methodological practice rather than a personal disclosure exercise. Ethnographic knowledge is co-produced through relationships, power dynamics, positional differences, language, access, and institutional context (McCorkel and Myers, 2003; Folkes, 2023). In this study, access to performers, cultural organizers, and officials developed gradually through repeated visits and sustained engagement. Participants may have framed their accounts differently depending on whether they were speaking as local practitioners, recognized inheritors, cultural officials, organizers, or community spectators.

Reflexive memos were maintained to document moments of hesitation, discomfort, misunderstanding, heightened rapport, and possible social desirability. These moments were treated analytically because they helped identify which perspectives were foregrounded, which claims required cautious interpretation, and where tensions existed between official narratives of safeguarding and local accounts of participation, humor, and authenticity. Reflexivity was particularly important because the study examines heritage governance, where symbolic authority, institutional legitimacy, and cultural ownership are central to the research problem.

### 2.8 Ethical considerations

This research received ethical approval from the Human Research Ethics Committee of Universiti Pendidikan Sultan Idris, Malaysia. The approved project was titled An Ethnographic Study of The Evolution of Quanfeng Huadeng from Small-scale Folk Opera to Intangible Cultural Heritage in Jiangxi Province, China. The approval reference number is 2025-0661-01, with an approval period from 28 February 2025 to 27 February 2026. The formal doctoral fieldwork conducted from March 2025 onward fell within this approval period.

Materials collected between 2021 and 2024 were treated as preliminary field engagement and contextual materials. They were used to establish historical background, identify key participants, refine the research questions, and support later verification. Earlier materials were included in the analysis only where consent, verification, anonymisation, and ethical conditions permitted their use. Direct quotations are used only where participants consented to the use of their interview materials for academic research and publication.

All participants involved in the formal doctoral fieldwork provided informed consent before interviews and observations. Participants were informed about the purpose of the study, voluntary participation, confidentiality procedures, and their right to withdraw. To preserve anonymity, participants are identified by role descriptors, participant codes, or pseudonyms where appropriate. Some named participants are identified only where they provided consent and where the information relates to public cultural roles or non-sensitive factual matters. The photographs included in this article have been anonymised by blurring potentially identifiable human faces and other visual identifiers where necessary. No unblurred identifiable facial images are published.

## 3 Results

This section presents findings from phased ethnographic engagement with Quanfeng Huadeng, with the principal evidence drawn from formal doctoral fieldwork and supported by verified preliminary and Supplementary material. The analysis is based on interviews with 21 core participants, approximately 30 brief audience interviews, group conversations and immediate audience feedback records, participant observation, documentary materials, field notes, photographs, and audio-visual recordings. The findings are organized around four interrelated dimensions of performance ecology: rhythm, spatial form, performer composition, and aesthetic orientation. These dimensions were developed through iterative thematic analysis and cross-material comparison across interviews, observation records, visual materials, and documentary sources. They are not treated as separate variables, but as connected dimensions through which heritage governance reshapes the conditions of performance, participation, improvisation, and local meaning-making.

### 3.1 Rhythm: from flexible village temporality to programmed performance time

The first theme concerns the transformation of performance rhythm. In village-based Huadeng, especially during Chinese New Year household and processional performances, time was organized according to festive atmosphere, host-family context, audience response, and performer judgement. Performers could extend or shorten a scene, repeat a section, pause for laughter, or adjust jiangbai according to the audience's mood. In contrast, institutionally organized performances were increasingly shaped by programme schedules, fixed duration, rehearsal requirements, and thematic expectations.

This pattern was developed through comparison of performer interviews, field notes, and video records from village-based and staged contexts. P6, a Sheng performer, described the difference clearly: in village settings, a Huadeng song could last more than 10 min and sometimes much longer, while staged performances were often limited to approximately 8 min. P6 explained that this shorter duration made it difficult to fully express the emotional meaning of the song. P9, a performer-organizer, similarly noted that when specialized instructors guided rehearsals, performers were required to adjust music, movement, formation, and lyrics. When performers organized the performance themselves, they were more likely to retain the original lyrics and revise mainly the jiangbai to suit the situation.

Observation records supported these accounts. In village performances, rhythm was flexible and interactional. Performers responded to laughter, verbal comments, and the social atmosphere of the host household. By contrast, staged performances moved through a more compressed sequence. Songs were excerpted, jiangbai was shortened, and transitions were planned according to programme requirements. This did not simply reduce performance length; it changed the conditions through which audience-responsive humor, narrative elaboration, and improvisation could occur.

The temporal transformation therefore shows how heritage governance reorganizes performance practice at the level of rhythm. Formal scheduling enabled Huadeng to enter festivals, publicity events, school activities, and cultural tourism programmes. At the same time, it reduced the temporal flexibility that had allowed performers and audiences to co-produce meaning during village-based performances. The shift is therefore best understood not as the disappearance of rhythm, but as a movement from socially negotiated festive temporality toward programmed performance time.

### 3.2 Spatial form and visual evidence: from co-present social space to staged separation

The second theme concerns spatial transformation. Visual evidence, observation records, and participant accounts show that Huadeng has increasingly moved from shared village spaces toward more formalized performance arrangements. Traditionally, Huadeng was performed in village entrances, courtyards, household halls, tangqian spaces, and open communal areas. These settings allowed performers and audiences to occupy the same physical and social environment. Audience members could stand close to the performers, laugh, comment, correct lyrics, move around, or respond directly to comic monolog. The three photographs included in this section are used as visual ethnographic evidence rather than decorative illustrations. They were selected because they show three contrasting spatial configurations: village-based co-present performance, transitional household performance, and institutionally organized staged or rehearsal-based performance.

Figure 1 shows a co-present village performance space. Performers and audience members share the same interior environment, with no raised stage or fixed seating boundary. The audience surrounds the performance area, and performers remain close enough for direct eye contact and spontaneous verbal exchange. This arrangement supports a relational mode of performance in which humor, movement, and audience response remain mutually visible.

Figure 2 represents a transitional spatial configuration. The performance still takes place inside a household setting, but the clearer open floor area and more directional orientation begin to distinguish between performance space and viewing space. Compared with Figure 1, the audience appears more positioned as viewers, although proximity is still maintained. This suggests that spatial transformation does not occur as a sudden break between “village” and “stage.” Rather, it develops gradually through changes in housing forms, audience habits, and performance expectations.

Figure 3 shows a more institutionalized spatial arrangement. Performers are organized in formal formation, oriented toward an external viewing position, and arranged for visual coordination. Compared with Figures 1, 2, this setting privileges visibility, symmetry, and presentational clarity. Interaction is less emergent and more pre-structured.

Field notes further confirmed this shift. In village settings, audience members could interrupt, comment, or respond directly, while performers adjusted delivery accordingly. In staged settings, lighting, sound systems, fixed viewing positions, and audience boundaries created a clearer division between performers and spectators. P7, a Sheng/Dan performer, described village performance as easier to adapt because performers could see and read the audience directly. In staged settings, performers had to project outward and follow the planned structure rather than respond continuously to spectators.

This does not mean that staged audiences were disengaged. Rather, their engagement was organized differently. Village-based engagement was more interventionist and relational, while staged engagement was more observational and spectatorial. The spatial transformation therefore changed the affordances of participation: it reduced opportunities for immediate verbal exchange but increased visual clarity and presentational order.

### 3.3 Performer composition: from community-embedded roles to managed participation

The third theme concerns changes in performer composition and organization. Earlier Huadeng performance depended heavily on local villagers, family networks, shared dialect, and long-term familiarity with local customs. Performers were not only role-bearers but also community members who understood the social situations of host families, local jokes, appropriate blessings, and the timing of improvised jiangbai. This embedded knowledge allowed performers to adapt repertoire and humor to specific households and audiences.

After heritage recognition, performer organization became more formalized. P1, a representative inheritor and cultural official, described how training sessions, rehearsal arrangements, and official performance tasks became more important after Huadeng entered the ICH system. Field records also show that the Quanfeng Village Cultural Center Huadeng Team mainly undertakes government-organized performances, while the Longquanduan Huadeng Team remains more closely associated with experienced village-based performance practice. This contrast provided an important basis for comparing institutional and community-embedded performance contexts.

The data also shows a shift toward managed participation. Cultural officials and troupe organizers increasingly coordinate rehearsals, performance opportunities, costumes, stage arrangements, and programme themes. In some cases, performers who previously appeared mainly during the Lunar New Year are now expected to participate in year-round cultural events. P1 described this as a move toward semi-professional management. Performer interviews and field notes also indicate that substitute or externally invited performers may be used when local performers are unavailable.

This development has both enabling and constraining effects. On the one hand, institutional coordination supports continuity by providing rehearsal opportunities, funding, costumes, visibility, and new performance platforms. It can also create opportunities for students, younger performers, women, and semi-professional participants to become involved in Huadeng. P5, a performer and organizer, described adapting Welcoming the Top Scholar (Jie Zhuangyuan 接状元) into a children's version for primary school students because some original pitches and movements were too difficult for young learners. This shows that institutional and educational settings can generate new pathways for transmission.

On the other hand, managed participation may weaken the local knowledge embedded in village performance. Technical skills, stage discipline, and costume coordination do not automatically reproduce dialect humor, household-specific references, or audience-sensitive jiangbai. P6 and P9 both suggested that performances organized for external tasks often required different priorities from performances organized for local audiences. The issue is therefore not whether institutional performers should be rejected, but how performer training can preserve local knowledge alongside stage technique.

The changing composition of performers thus redistributes agency. In village performance, agency was grounded in local familiarity and improvisational authority. In staged performance, agency is increasingly shaped by rehearsals, institutional expectations, role allocation, programme requirements, and public display.

### 3.4 Aesthetic orientation: from participatory wit to curated display

The fourth theme concerns aesthetic orientation. Village-based Huadeng valued verbal wit, local dialect, flexible jiangbai, live gong-and-drum cues, comic timing, and immediate audience response. Success depended not only on visual presentation, but on whether the performance could activate shared laughter, moral recognition, social memory, and local familiarity.

In institutional and staged contexts, aesthetic value increasingly shifted toward costume coordination, choreographed movement, amplified sound, subtitles, stage formation, thematic clarity, and visual polish. This pattern emerged through comparison of performer interviews, audience feedback records, observation notes, and video records. P2, a senior representative inheritor, expressed concern that some contemporary staged versions retained the outward appearance of Huadeng while weakening its older interactive qualities. P5, by contrast, emphasized that adaptation was necessary because younger learners and non-local audiences may not understand older songs and local expressions without adjustment.

This tension was especially visible in staged performances designed for non-local audiences. Field notes from a cultural tourism performance recorded that Going to Nanjing (Xia Nanjing 下南京) was condensed into a short stage segment, performed with uniform costumes, introduced by a host, and presented to an audience located in a designated viewing area. Audience interaction was limited, and applause occurred mainly at planned points in the programme. This format improved clarity and manageability, but it differed significantly from the interactional rhythm of village-based performance.

Subtitles and spoken introductions also produced mixed effects. They helped audiences unfamiliar with the local dialect follow the storyline, but they sometimes disrupted comic timing. P5 noted that in one staged performance, the subtitles moved too quickly and revealed the ending before the performer delivered the punchline, causing the audience to stop laughing. This example shows that accessibility tools can support comprehension while also changing the timing, suspense, and humor on which Huadeng depends.

Audience feedback records reinforced this tension. Some audience members valued staged performances because they appeared more orderly, colorful, and easier to understand. Others felt that staged Huadeng sounded less natural than household performances and described it as closer to recitation than spontaneous performance. These responses should not be treated as a simple opposition between “authentic village” and “inauthentic stage.” Rather, they show that different performance contexts produce different criteria of value. Village audiences often valued familiarity, dialect humor, and direct interaction, while staged audiences were more likely to evaluate visual clarity, coordination, and accessibility.

From the perspective of performance ecology, aesthetic formalization changes the conditions of engagement. In village settings, engagement often involved laughter, verbal correction, movement, direct response, and recognition of local references. In staged settings, engagement was more often organized around watching, listening, photographing, applauding, and interpreting Huadeng as cultural representation. The shift is therefore not from engagement to non-engagement, but from one mode of engagement to another.

### 3.5 Synthesis: a dual performance ecology

Across the four themes, the findings show that Quanfeng Huadeng has developed into a dual performance ecology. Village-based Huadeng continues to value flexible rhythm, spatial proximity, local dialect, household relations, improvised jiangbai, and audience-responsive humor. Institutionally staged Huadeng values programme order, visual clarity, coordinated formation, wider communicability, and public cultural representation.

This dual ecology is not a simple division between tradition and modernity. Practitioners move between both contexts and adjust their performance strategies accordingly. The same performer may preserve a longer and more flexible style in village contexts while accepting shortened excerpts, revised lyrics, and formal formations in staged settings. Heritage governance has therefore not simply erased local practice. It has reorganized the rules, resources, and expectations through which practice is enacted. Table 1 summarizes how the four analytical themes were developed through cross-material comparison.

**Table 1:** Cross-material synthesis of performance ecology transformation in Quanfeng Huadeng.

Analytical dimension	Main evidence sources	Village-based performance ecology	Institutionally staged performance ecology	Analytical implication for participation, agency, and engagement
Rhythm	P6/P9 interviews; FN; VR	Flexible duration; pacing shaped by audience response and household context	Shortened programme slots; fixed sequence; time discipline	Improvisation becomes more constrained because there is less time for narrative extension, audience-responsive humor, and spontaneous performer adjustment
Spatial form	Figures 1-3; FN; A records	Shared space; close proximity; direct eye contact; verbal exchange	Stage orientation; audience separation; formal viewing position	Participation shifts from interventionist and co-present response toward more observational and spectatorial engagement
Performer composition	P1/P5/P6/P9 interviews; rehearsal observation; documentary materials	Local villagers; shared dialect; kinship and community knowledge	Managed roles; training systems; occasional substitute or semi-professional participation	Institutional systems create new transmission opportunities, but local contextual knowledge may weaken if not supported by dialect, village-based practice, and improvisational training
Aesthetic orientation	P2/P5 interviews; A records; FN; VR	Verbal wit, *jiangbai*, live cues, humor, situational adaptation	Costumes, choreography, subtitles, amplified sound, visual clarity	Aesthetic value shifts from participatory humor and local recognition toward visual clarity, accessibility, and cultural representation

P, core interview participant; A, brief audience interview, group conversation, or immediate audience feedback record; FN, field notes; VR, video record. Figures 1-3 refers to selected visual ethnographic evidence. The table summarizes how the four analytical dimensions were developed through comparison of interviews, field observations, audience feedback, visual materials, documentary sources, and audio-visual records.

## 4 Discussion

This study examined how heritage governance reconfigures the performance ecology of Quanfeng Huadeng. The findings show that Huadeng has not simply been preserved, lost, or replaced. Rather, it now operates within a dual performance ecology in which village-based festive practice and institutionally staged cultural presentation coexist. This duality is analytically important because it reveals that heritage transformation occurs not only through changes in repertoire, costume, or institutional status, but also through changes in the psychological and interactional conditions under which performance is perceived, enacted, and made meaningful.

The results contribute to three bodies of scholarship. First, they extend intangible cultural heritage research by showing how heritage governance reorganizes live performance at the level of rhythm, space, performer composition, and aesthetic value. Second, they contribute to cultural and ecological psychology by demonstrating how engagement is shaped by situated affordances such as proximity, visibility, timing, audience familiarity, and opportunities for response. Third, they refine discussions of agency by showing that performer agency does not disappear under institutionalization, but becomes redistributed across rules, resources, venues, audiences, and performance expectations.

### 4.1 Beyond preservation or loss: heritage governance as ecological reorganization

Existing heritage scholarship has shown that official recognition often transforms living practices by making them more visible, legible, and governable (Kirshenblatt-Gimblett, 2004; Norton and Matsumoto, 2018; Zhu, 2012). The findings of this study are consistent with that argument, but they also extend it. In Quanfeng Huadeng, heritagisation does not merely produce a new public image of the tradition; it reorganizes the performance ecology through which the tradition functions. Fixed performance times, stage arrangements, training systems, thematic requirements, costumes, subtitles, and audience boundaries all reshape what performers and audiences can do during performance.

This finding is important because many discussions of ICH governance focus on institutional categories, policy frameworks, or questions of authenticity. The Huadeng case shows that the effects of governance are also micro-interactional. A shortened programme does not only reduce duration; it narrows the time available for improvisation. A raised stage does not only change visibility; it changes eye contact, bodily orientation, and the possibility of audience interruption. A substitute or semi-professional performer does not only fill a role; they may change the local knowledge available within the performance. In this sense, the study shifts attention from heritage as a preserved object to heritage as an organized field of interaction.

This also complicates a simple critique of heritage governance. The findings do not support the view that institutional intervention only damages local tradition. Heritage governance has provided Huadeng with visibility, rehearsal opportunities, official recognition, training mechanisms, and new transmission platforms. These resources matter because the village-based social base of Huadeng has already been weakened by aging performers, youth migration, urbanization, and changing entertainment habits. However, the same mechanisms that sustain visibility may also standardize the conditions of performance. The central issue is therefore not whether governance is good or bad, but how its rules and resources reshape the relational conditions through which living heritage remains meaningful.

### 4.2 Performance ecology and situated engagement

For cultural and ecological psychology, the findings show that engagement is not only an internal state of the individual. It is shaped by the affordances of the performance environment. Village-based Huadeng offers affordances for proximity, eye contact, verbal response, humor, correction, movement, and performer adaptation. Staged Huadeng offers different affordances: visibility, order, projection, visual appreciation, documentation, and cultural representation. Both contexts can produce engagement, but they organize engagement differently.

This point refines the article's use of psychological engagement. Rather than claiming that staged performances simply reduce engagement, the findings suggest a shift in the mode of engagement. In village settings, engagement is often behavioral and relational: spectators laugh, interrupt, correct, move, comment, and become part of the performance situation. Emotional engagement is supported by familiarity, local dialect, household relations, nostalgia, humor, and shared memory. Cognitive engagement emerges through recognition of local references, interpretation of improvised jiangbai, and understanding of social situations embedded in the performance. In staged settings, engagement is more spectatorial: audiences watch, listen, photograph, applaud, and interpret Huadeng as cultural display.

This distinction is especially relevant for Frontiers in Psychology because it positions engagement as a situated and culturally mediated process. The study does not treat behavioral, emotional, and cognitive engagement as psychometric variables measured by scale. Instead, it examines how the environment makes certain forms of participation more or less available. This approach is consistent with cultural psychology, which emphasizes that meaning and identity are mediated through culturally organized practices (Miller and Chauhan, 2012; Valsiner, 2013; Wan and Chew, 2013), and with ecological perspectives that view environments as shaping perception, action, and participation (De Young, 2013; Kals et al., 2025). The Huadeng case therefore demonstrates how cultural performance can be understood as a psychological ecology: a setting in which bodies, spaces, sounds, memories, roles, and social expectations jointly organize experience.

### 4.3 Rhythm, improvisation, and the psychology of timing

The transformation of rhythm provides a concrete example of how heritage governance affects situated cognition and creativity. In village contexts, performers adjust the pace of performance according to audience mood, household setting, laughter, and social occasion. This supports improvisational decision-making. In staged contexts, performance time is increasingly governed by programme schedules and fixed duration. Longer narrative and comic sequences are condensed, while jiangbai and musical sections are selected or shortened.

This finding supports scholarship on improvisation as an interactional and socially coordinated process rather than an isolated act of individual creativity (Kenny and Gellrich, 2002; Macdonald and Wilson, 2020; Sawyer, 2003). In Huadeng, improvisation depends on shared repertoire, local dialect, performer memory, comic timing, and audience response. When the performance is compressed into a short stage slot, performers may still exercise agency, but the temporal affordances for improvisation are reduced. The result is not the disappearance of creativity, but a change in the conditions under which creativity can occur.

This finding also contributes to psychological discussions of timing and interaction. In village Huadeng, timing is relational: pauses, repetitions, laughter, and responses are part of a shared temporal field. In staged Huadeng, timing becomes more programmatic: transitions, entrances, exits, and endings are coordinated according to an external event structure. The shift from relational timing to programmed timing changes how performers monitor audiences, how spectators anticipate humor, and how shared affect develops. Thus, rhythm becomes a psychological as well as an aesthetic issue.

### 4.4 Space, embodiment, and participation

The findings on spatial form extend existing heritage studies by showing how spatial reconfiguration alters embodied participation. Previous research on heritage performance and tourism has shown that cultural practices may become more staged, displayable, and visitor-oriented (Zhu, 2012; Chen et al., 2021; Maags, 2021; Su, 2023). The Huadeng case adds a more fine-grained account of how this process works in performance. Spatial distance changes what performers can perceive and what audiences can do. In village spaces, performers can see faces, hear comments, and respond to bodily cues. Audiences can move, laugh, interrupt, and correct. In staged spaces, performers face a more generalized audience, while spectators are positioned as viewers.

This contributes to ecological psychology by showing that performance spaces are not passive containers. They structure perception-action possibilities. A household hall, a village courtyard, and a stage do not simply host the same performance in different locations; they produce different possibilities for attention, movement, response, and social recognition. In Huadeng, the shift from co-present space to staged separation therefore changes not only the visual form of performance but also the embodied relationship between performer and spectator.

This insight is important because psychological engagement in performance is often discussed through individual response, preference, or affect. The Huadeng case suggests that engagement must also be analyzed through spatial arrangements. Who can see whom? Who can respond? Who can interrupt? Who is allowed to move? Who controls the direction of attention? These questions show how spatial design shapes participation before any individual response occurs.

### 4.5 Performer composition and the redistribution of agency

The findings also complicate assumptions about community agency. In some heritage debates, community-based performance is treated as locally authentic, while institutional performance is treated as externally imposed. The Huadeng case shows a more complex situation. Institutional systems can weaken certain forms of local knowledge, but they can also create new opportunities for transmission. Training programmes, school-based adaptations, public performances, and semi-professional participation may allow younger performers, women, students, and non-core villagers to participate in ways that were not previously available.

This finding is broadly consistent with studies of institutionalized Chinese performing traditions, which show that formal organizational systems can support continuity while reshaping performer roles and cultural authority (Yang and Wu, 2026). However, the Huadeng case adds a more specific psychological and interactional dimension. Performer substitution or professionalization does not only change personnel. It changes the knowledge available in performance. Dialect humor, household references, improvised blessings, and audience-sensitive timing are forms of situated knowledge. They cannot be transmitted fully through choreography or script alone.

Agency is therefore redistributed rather than simply reduced. In village settings, agency is grounded in local familiarity, oral memory, and improvisational authority. In staged settings, agency is shaped by rehearsal systems, official themes, stage rules, audience expectations, and institutional evaluation. Performers may still make choices, but those choices are made within a different ecology of constraint and possibility. This provides a more nuanced account of agency than a simple opposition between local autonomy and institutional control.

### 4.6 Aesthetic formalization and the transformation of value

The results also speak to debates on heritage value. In village-based Huadeng, aesthetic value is tied to humor, timing, dialect, audience response, and the performer's ability to adapt to local context. In staged Huadeng, value is increasingly tied to clarity, visual polish, costume coordination, stage order, subtitles, and communicability to wider audiences. This supports Kirshenblatt-Gimblett (2004) argument that heritage processes often reorganize cultural practices according to exhibitionary and representational logics. It also aligns with studies showing that heritage and tourism development can increase visibility while reshaping local meanings and evaluative criteria (Zhu, 2012; Chen et al., 2021; Maags, 2021; Su, 2023).

However, the Huadeng case also shows that aesthetic formalization is not merely superficial. It changes how audiences understand and evaluate the performance. Subtitles may improve comprehension for non-local audiences, but they may also reveal comic turns too early and weaken humor. Uniform costumes may enhance visual coherence, but they may also shift attention away from verbal improvisation. Shorter scripts may make the performance easier to programme, but they may reduce narrative depth. These findings show that aesthetic changes have psychological consequences because they reshape attention, expectation, surprise, humor, and emotional resonance.

This is where the study contributes to a psychology-oriented understanding of heritage performance. Aesthetic form is not only a cultural surface; it organizes experience. Stage polish, subtitles, lighting, sound, movement, and timing affect how audiences attend, anticipate, interpret, and respond. This point is consistent with cultural psychology's view that meaning is mediated through culturally organized practices (Miller and Chauhan, 2012; Valsiner, 2013; Wan and Chew, 2013), and with research on musical engagement as situated, affective, and socially embedded (MacDonald and Saarikallio, 2022). The transformation of Huadeng therefore illustrates how cultural governance can alter the psychology of reception by changing the material and social design of performance.

### 4.7 Rethinking safeguarding: from cultural form to participatory conditions

A key implication of the study is that safeguarding should not be limited to preserving repertoire, costume, scripts, and staged versions. These elements are important, but they do not preserve the performance ecology of Huadeng by themselves. Cultural vitality also depends on temporal flexibility, spatial proximity, dialect practice, audience response, household-based performance, improvisational competence, and local performer knowledge. This argument extends heritage scholarship that warns against treating living traditions as stable objects to be documented, displayed, or standardized (Kirshenblatt-Gimblett, 2004; Zhu, 2012; Norton and Matsumoto, 2018).

This argument does not reject institutional safeguarding. Official performances, festivals, training programmes, documentation projects, and tourism events are important for public recognition and continuity. In China, ICH governance has provided crucial mechanisms for recognition, transmission, representative inheritor support, and local cultural development (Li et al., 2025; Zhu, 2026). The problem arises when staged forms become the dominant standard by which the tradition is judged. If safeguarding privileges only what is visible, teachable, displayable, and programmatically efficient, it may preserve the appearance of Huadeng while weakening the conditions that historically sustained its local meaning.

A more ecologically informed approach to safeguarding would support plural modes of practice. Village-based performances, household performances, processional performances, school-based learning, staged showcases, and tourism presentations can coexist, but each should be evaluated according to its function. Village-based contexts should not be treated as informal or secondary simply because they are less polished. They are crucial sites for sustaining humor, audience response, dialect, memory, and social connection. Similarly, staged contexts should not be dismissed as inauthentic, because they provide visibility, resources, and new publics. This balanced view is consistent with studies showing that institutionalized performance systems can support continuity while also reshaping cultural authority and performer roles (Yang and Wu, 2026). The challenge is to design safeguarding practices that allow these ecologies to interact without reducing one to the other.

### 4.8 Theoretical contribution: performance ecology transformation

The concept of performance ecology transformation is the main theoretical contribution of this study. It explains how heritage governance affects not only the external form of a tradition but also the relational system through which performance becomes meaningful. In Quanfeng Huadeng, rhythm, space, performer composition, and aesthetic orientation are not separate features. They interact. A shorter performance time reduces improvisational space; a staged spatial arrangement changes audience response; a different performer composition affects dialect humor and local knowledge; aesthetic formalization changes attention and evaluation. This relational view extends heritage studies by moving beyond a narrow concern with preservation, authenticity, or cultural display (Kirshenblatt-Gimblett, 2004; Zhu, 2012; Norton and Matsumoto, 2018).

The concept also contributes to cultural and ecological psychology. Cultural psychology emphasizes that identity, meaning, and social relations are mediated through culturally organized practices (Miller and Chauhan, 2012; Valsiner, 2013; Wan and Chew, 2013). Ecological perspectives further suggest that environments structure what forms of perception, action, and participation become possible (De Young, 2013; Kals et al., 2025). The Huadeng case brings these perspectives together by showing how engagement, agency, and meaning making are distributed across people, spaces, bodies, sounds, institutional rules, and material arrangements.

Performance ecology transformation therefore provides a way to analyse living heritage without reducing change to either cultural loss or successful preservation. It highlights how institutional rules and resources are negotiated in situated practice, consistent with structuration theory's view that structure both enables and constrains action (Giddens, 1984). It also clarifies why improvisation, participation, and engagement must be analyzed as situated processes shaped by time, space, audience familiarity, and social coordination (Kenny and Gellrich, 2002; Sawyer, 2003; MacDonald and Saarikallio, 2022). The concept may therefore be useful for studying other living heritage traditions that move between community-based contexts and staged public representation.

### 4.9 Limitations and future research

This study is based on a single ethnographic case of Quanfeng Huadeng and should not be generalized uncritically to all Chinese intangible cultural heritage practices or folk opera traditions. The analysis is interpretive and draws on interviews, observation, field notes, documentary materials, photographs, and audio-visual records. Claims about participation, agency, and engagement are therefore grounded in ethnographic evidence and participant accounts rather than direct psychological measurement.

The fieldwork involved phased engagement across different periods, including preliminary field engagement, formal doctoral fieldwork, and supplementary verification. This long-term engagement strengthened contextual understanding, but it also required careful distinction between contextual materials and formally verified data. In addition, the study focuses mainly on performers, organizers, officials, research association members, and audience members accessible through the researcher's field relationships. The perspectives of younger non-participants, migrant villagers, audiences who no longer attend Huadeng performances, and policy actors beyond the local level are less visible.

Future research could compare Quanfeng Huadeng with other Huadeng traditions or small-scale folk operas in China to examine whether similar performance ecology transformations occur elsewhere. Psychology-oriented research could also examine audience experience more directly through interviews, observation, or mixed methods focused on attention, affect, memory, and participation across village-based and staged contexts. Such work would further clarify how different performance ecologies shape the psychological experience of living heritage.

## 5 Conclusion

This study examined how heritage governance has reconfigured the performance ecology of Quanfeng Huadeng, a small-scale folk opera from Xiushui County, Jiangxi Province. Drawing on ethnographic interviews, participant observation, field notes, documentary materials, visual evidence, and audio-visual records, the study showed that Huadeng has not simply moved from “authentic village tradition” to “inauthentic staged display.” Rather, it now operates within a dual performance ecology in which village-based festive practice and institutionally staged cultural presentation coexist.

The findings answer the three research questions by showing that heritage governance has reshaped Huadeng through four interrelated dimensions: rhythm, spatial form, performer composition, and aesthetic orientation. Performance time has become more compressed and programme-oriented; performance space has shifted from co-present village settings toward more separated staged arrangements; performer participation has moved from community-embedded roles toward more managed and semi-professional forms; and aesthetic value has increasingly emphasized visual clarity, stage order, accessibility, and cultural representation. These transformations have brought visibility, resources, and new transmission opportunities, but they have also changed the conditions through which improvisation, dialect humor, audience response, and local performer agency are enacted.

The study contributes to intangible cultural heritage research by arguing that safeguarding should not be understood only as the preservation of repertoire, costumes, scripts, or staged forms. For living performance traditions such as Quanfeng Huadeng, cultural vitality also depends on the social, spatial, temporal, and participatory conditions that allow performance to remain meaningful in local life. This means that household performances, village processions, improvised jiangbai, dialect humor, audience correction, and performer–spectator proximity should not be treated as secondary or informal elements. They are part of the performance ecology through which Huadeng becomes socially and culturally significant.

The study also contributes to psychology-oriented discussions of cultural performance by showing that engagement, agency, and meaning-making are situated rather than purely individual processes. Audience engagement in Huadeng is shaped by spatial proximity, timing, humor, familiarity, bodily orientation, sound, visibility, and opportunities for response. Staged performances do not necessarily eliminate engagement, but they reorganize it from a more relational and interventionist mode toward a more spectatorial and representational mode. This distinction is important because it shows how cultural governance can alter the psychological and interactional experience of performance by changing the material and social design of the performance environment.

The concept of performance ecology transformation offers a case-based analytical tool for understanding this process. It helps explain how institutional rules and resources affect not only what is performed, but how performance becomes possible, meaningful, and emotionally resonant. In the case of Quanfeng Huadeng, rhythm, space, performer composition, and aesthetic form are not separate features; they interact to shape the affordances of participation, improvisation, attention, and cultural interpretation.

The study is limited by its focus on a single ethnographic case and by its interpretive rather than psychometric treatment of engagement. Its claims should therefore not be generalized uncritically to all Chinese intangible cultural heritage practices or folk opera traditions. Nevertheless, the case provides a detailed account of how living heritage is transformed when community-based performance enters institutional systems of safeguarding, display, and cultural development. Future research could compare Quanfeng Huadeng with other regional performance traditions and examine more directly how different performance ecologies shape audience attention, affect, memory, and participation.

Overall, the study suggests that sustainable safeguarding requires more than keeping a tradition visible. It requires protecting the conditions that allow people to perform, respond, remember, adapt, and make meaning together. For Quanfeng Huadeng, the future of the tradition depends not only on whether it continues to appear on stage, but also on whether its village-based, participatory, humorous, and improvisational ecology remains alive.

## Data Availability

The datasets presented in this article are not readily available because The dataset contains interview transcripts, field notes, audio-visual recordings, and visual materials from ethnographic research involving human participants in a small community. These materials are not publicly available due to ethical restrictions, participant confidentiality, and the risk of indirect identification. Identifiable visual materials and full raw data cannot be shared. An anonymised methodological audit trail is provided as Supplementary File 1. Anonymised excerpts or summary materials may be considered by the corresponding author upon reasonable request, where ethically permissible Requests to access the datasets should be directed to Lu Liang, luliangphd@163.com.
