# Supplementary material for: Tracing the reconfiguration of performance ecology in Quanfeng Huadeng: an ethnographic study of heritage governance, agency, and participation
Source: Front Psychol. 2026 Aug 11;17:1866204. doi: 10.3389/fpsyg.2026.1866204 (PMC13503160; doi:10.3389/fpsyg.2026.1866204)
Supplement: Supplementary file 1 [file Table_1.docx]

**Supplementary File 1. Anonymised Methodological Audit Trail**

**Manuscript title:** Tracing the Reconfiguration of Performance Ecology in Quanfeng Huadeng: An Ethnographic Study of Heritage Governance, Agency, and Participation

**Purpose of this supplementary file:** This document provides an anonymised audit trail of the ethnographic corpus, participant coding, analytical coding, triangulation procedure, and ethical safeguards used in the manuscript. It contains summary matrices only. It does not include raw transcripts, audio/video files, identifiable photographs, full field notes, participant contact details, household addresses, or unredacted personal information.

**Confidentiality note:** Core participants are coded as P1-P21. Brief audience interviews, group conversations, and immediate audience feedback records are coded as A1-A30. Field notes are coded as FN, video records as VR, audio records as AR, and photographs as Photo 1-3. The codes are used only to support analytical traceability and are not intended to reveal participant identity.

# S1. Fieldwork overview and analytical use

**Table S1a. Preliminary and contextual field engagement (2021-2024).**

| **Time** | **Fieldwork stage** | **Broad setting** | **Main activities** | **Performances / activities observed** | **Analytical use** | **Ethics status** |
| --- | --- | --- | --- | --- | --- | --- |
| February 2021 | Preliminary contextual engagement | Quanfeng Village; Chinese New Year settings | Initial interviews; observation of Chinese New Year performances | Approx. 10 Chinese New Year performances observed | Historical context; performance ecology; comparison of pre/post-2006 recollections | Used only where consent, verification, anonymisation, and ethical conditions permitted |
| August 2021 | Preliminary contextual engagement | Quanfeng Village | Initial interviews; repertoire and local documentary collection | Data collection outside Chinese New Year period | Repertoire, local terminology, performer memory, musical/monologue materials | Contextual and verification use |
| January-February 2022 | Preliminary fieldwork | Village households and processional settings | Observation of household and processional performances; informal interviews | Five household performances observed | Village-based spatial arrangements, performer-audience interaction, audience response | Contextual and verification use |
| October 2022 | Preliminary fieldwork | Central square / public event setting | Observation of formal staged performance context | Formal public/official performance observed | Comparison with village performance setting | Contextual and verification use |
| January 2023 | Preliminary fieldwork | Village households and local cultural settings | Interviews with inheritors/senior performers; documentary verification | Approx. 10 Chinese New Year performances observed | Oral history, ICH institutional context, performer memory | Contextual and verification use |
| February 2024 | Preliminary doctoral field engagement | Village households, routes, open spaces | Observation of Chinese New Year household and processional performances; audience feedback records | Approx. 40-50 household/processional performances observed | Audience interaction, jiangbai, rhythmic flexibility, spatial proximity | Contextual and verification use |
| September 2024 | Preliminary doctoral field engagement | Central square / National Day context | Observation of formal staged performances | Formal staged performances observed | Staged spatial arrangement, programme timing, visual presentation | Contextual and verification use |

**Table S1b. Formal doctoral fieldwork and supplementary verification (2025-2026).**

| **Time** | **Fieldwork stage** | **Broad setting** | **Main activities** | **Performances / activities observed** | **Analytical use** | **Ethics status** |
| --- | --- | --- | --- | --- | --- | --- |
| March-August 2025 | Formal doctoral fieldwork | Quanfeng Village, Quanfeng Township, Xiushui County | Systematic interviews, participant observation, verification of earlier materials, documentary/audiovisual collection | Multiple village-based, rehearsal, publicity, and staged performance contexts | Principal evidence for article analysis and three research questions | Main data period under ethics approval |
| May 2025 | Key formal case within doctoral fieldwork | Xigang Town / public performance setting | Observation of Labour Day Opera-to-the-Countryside performance | Formal staged/public performance | Programme format, staging, audience positioning, public cultural display | Main analytical corpus |
| August 2025 | Key formal case within doctoral fieldwork | Quanfeng Village open space / public square | Observation of legal-publicity performance and rehearsal/preparation | Policy-themed staged performance | Spatial formalisation, thematic adjustment, performer organisation | Main analytical corpus |
| October 2025 | Supplementary verification | Quanfeng / related field settings | Selective follow-up interviews and verification | Selective performance/context verification | Verification of coding and interpretations | Within ethics approval period |
| January 2026 | Supplementary verification | Central Square / Third Quanfeng Huadeng Arts Festival | Observation and on-site interviews | Formal arts festival context | Verification of staged performance ecology and public display | Within ethics approval period |

# S2. Participant category matrix

The counts below are role-category counts, not mutually exclusive statistical groups. A single participant may appear in more than one role category, for example as performer, representative inheritor, troupe organiser, or research association member. The core interview dataset comprises 21 participants.

| **Participant category** | **Count** | **Main data types** | **Principal topics** | **Analytical relevance** |
| --- | --- | --- | --- | --- |
| Senior performers / representative inheritors | 6 | Oral history interviews; semi-structured interviews | Memories of earlier performance practice; repertoire preservation; learning methods; early role experience | RQ1; historical and governance context |
| Sheng performers | 9 | Semi-structured interviews; observation; informal conversations | Monologue, repertoire selection, rhythmic control, differences between formal and informal performances | RQ3; rhythm, aesthetics, agency |
| Dan and Chou performers / gong-and-drum accompanists | 6 | Interviews; observation | Role allocation, gong-and-drum coordination, performance structure, adaptations for formal staged performances | Performance composition and aesthetic orientation |
| Huadeng troupe leaders / organisers | 2 | Semi-structured interviews; informal conversations | Troupe organisation, performance arrangements, training, coordination with government activities | RQ2; governance and organisation |
| Local cultural officials | 2 | Semi-structured interviews; documentary verification | ICH nomination process, representative inheritor system, funding, performance and training mechanisms | RQ2; heritage governance |
| Audience members | 5 | Informal interviews; records of immediate audience feedback | Evaluations of Chinese New Year performances, reception of formal staged performances, listening expectations, changing aesthetic preferences | RQ2-RQ3; audience reception |
| Members of Quanfeng Huadeng Research Association | 5 | Interviews; documentary verification | Compilation of documentary materials, safeguarding practices, local research, future development evaluations | Contextual verification and local knowledge |

# S3. Core participant code summary used for analytical traceability

This table lists the main coded participants cited or directly informing the article analysis. To reduce identifiability, age is presented as a broad age band in 2025 rather than an exact birth year, and no names or contact details are provided. Remaining core participants are grouped where further detail would risk over-identification or is not needed for the article evidence trail.

| **Code** | **Broad role** | **Gender** | **Age band** | **Type of data** | **Principal analytical contribution** |
| --- | --- | --- | --- | --- | --- |
| P1 | Representative inheritor / cultural official | Male | Approx. 70s | Semi-structured interviews | ICH system, training activities, nomination process, semi-professional management |
| P2 | Senior performer / representative inheritor | Male | Late 70s | Oral history interviews | Earlier performance memory; concern over staged transformation; role experience |
| P3 | Representative inheritor | Male | Approx. 70s | Semi-structured interviews | ICH system, training activities, staged performance experience |
| P4 | Senior Sheng performer | Male | Mid-80s | Interviews | Traditional repertoire, early performance practices, embodied memory |
| P5 | Sheng performer / organiser | Male | Early 60s | Interviews; observation | Formal/informal performance comparison; children/youth adaptation; staged accessibility |
| P6 | Sheng performer | Male | Late 50s | Interviews; observation | Monologue, rhythmic control, audience interaction, repertoire selection |
| P7 | Sheng/Dan performer | Male | Mid-50s | Interviews; observation | Role changes, monologue and humour, performance memory, spatial adaptation |
| P8 | Sheng performer | Male | Late 50s | Interviews; observation | Formal/informal performance comparison; performance memory |
| P9 | Chou performer / organiser | Male | Late 50s | Interviews; observation | Formal/informal comparison; monologue and humour; performance memory |
| P10 | Village audience member | Male | Approx. 90s | Interview | Audience reception, aesthetic evaluation, Chinese New Year performance feedback |
| P11 | Non-local audience member | Female | Mid-40s | Interview | Audience reception, staged-performance evaluation, accessibility |
| P12 | Research Association member | Male | Mid-50s | Interview | Documentary compilation, safeguarding practice, local evaluations |
| P13-P21 | Additional core participants across performer, audience, organiser, official, and local research categories | Mixed / anonymised | Anonymised | Interviews; informal conversations; verification | Contextual verification and supplementary triangulation; not individually detailed to reduce identifiability |

# S4. Audience feedback records

Audience records are not treated as 30 independent core participants. They summarise brief interviews, group conversations, and immediate responses recorded in village processional/household and formal staged settings. The records are used to support analysis of reception, listening expectations, humour, dialect comprehension, and perceived differences between village-based and staged performance.

| **Audience record code** | **Context** | **Data form** | **Main reception pattern** | **Analytical use** |
| --- | --- | --- | --- | --- |
| A1-A12 | Village household / processional performances | Immediate post-performance comments; informal group feedback | Familiarity, dialect humour, direct response, red-packet evaluation, correction of lyrics, household atmosphere | Spatial form; aesthetic orientation; engagement |
| A13-A20 | Village open-space / community festival settings | Brief audience conversations and observation notes | Crowd movement, laughter, comments, recognition of local references, comparison with earlier Huadeng memories | Rhythm; spatial form; audience reception |
| A21-A26 | Formal staged / publicity performances | Immediate feedback and observation notes | Visual order, costumes, official themes, clearer presentation, limited direct interaction | Aesthetic formalisation; staged engagement |
| A27-A30 | Tourism / non-local or mixed audience contexts | Brief audience comments and researcher notes | Subtitles, comprehension, accessibility, reduced local humour, spectatorial viewing | Accessibility; psychology of reception |

# S5. Coding tree: from initial codes to analytical themes

| **Initial codes / evidence indicators** | **Intermediate categories** | **Final analytical theme** | **Interpretive focus** |
| --- | --- | --- | --- |
| Chinese New Year processional performances; household performances; stage compression; programme duration; fixed time slots; pauses and laughter | Sound and rhythmic organisation; performance temporality | Rhythm: from flexible village temporality to programmed performance time | Shows how time discipline affects improvisation, narrative elaboration, and audience-responsive humour |
| Tangqian spaces; household halls; open village spaces; stage orientation; audience boundaries; body orientation; lighting/sound; performer-audience distance | Performance settings; visual/spatial organisation | Spatial form: from co-present social space to staged separation | Shows how spatial affordances reorganise participation and engagement |
| Representative inheritor system; troupe organisation; rehearsal requirements; substitute performers; youth/school adaptation; role allocation; semi-professional management | Institutional mechanisms; performer strategies | Performer composition: from community-embedded roles to managed participation | Shows how agency and cultural authority are redistributed |
| Jiangbai adaptation; subtitles; costumes; choreographed movement; official themes; local humour; live cues; dialect comprehension; audience evaluation | Aesthetic evaluation; audience reception; heritage representation | Aesthetic orientation: from participatory wit to curated display | Shows how aesthetic formalisation shifts attention, value, and reception |

# S6. Cross-material evidence matrix

| **Analytical theme** | **Main source types** | **Observed / reported pattern** | **Interpretive claim** |
| --- | --- | --- | --- |
| Rhythm | P6/P9 interviews; FN; VR | Village performances allowed extension, pauses, laughter, and situational adjustment; staged events required shorter and more fixed slots | Temporal compression interpreted as institutional time discipline and reduced space for improvisation |
| Spatial form | Photo 1-3; FN; A records; P7 interview | Village settings showed co-presence and direct response; staged settings showed distance, orientation, and clearer boundaries | Spatial change interpreted as altered affordances for participation and engagement |
| Performer composition | P1/P5/P6/P9 interviews; rehearsal observation; documentary materials | Institutional support created training and public platforms; managed participation and substitute performers changed local knowledge base | Performer agency interpreted as redistributed across rules, resources, and local familiarity |
| Aesthetic orientation | P2/P5 interviews; A records; FN; VR | Village value centred on humour, dialect, jiangbai, and live response; staged value centred on visual clarity, subtitles, costumes, and accessibility | Aesthetic formalisation interpreted as transformation of reception, attention, and heritage value |

# S7. Visual evidence audit trail

| **Visual code** | **Broad context** | **Spatial configuration** | **Analytical indicators** | **Ethics safeguard** |
| --- | --- | --- | --- | --- |
| Photo 1 | Chinese New Year 2025; traditional village residence main hall | Village-based co-present performance | Performer-audience proximity, absence of raised stage, shared interior environment, direct exchange | Used with explicit consent or anonymised where necessary; no additional location detail provided |
| Photo 2 | Chinese New Year 2024; newly constructed village house | Transitional household performance | Emerging separation between performance and viewing area while maintaining proximity | Used with explicit consent or anonymised where necessary; no household identity disclosed |
| Photo 3 | Legal-publicity rehearsal/performance preparation; county/public cultural context | Institutionally organised staged/rehearsal-based performance | Formal formation, external viewing orientation, visual coordination, reduced emergent interaction | Used with explicit consent or anonymised where necessary; exact date to be confirmed in manuscript |

# S8. Translation, verification, and interpretation procedure

| **Procedure element** | **Description** |
| --- | --- |
| Interview language | Mandarin, local Quanfeng dialect, or both, depending on participant and context |
| Translation pathway | Quanfeng dialect -> Standard Mandarin -> English for manuscript preparation |
| Terms retained | Key performance terms and repertoire titles retained in pinyin or Chinese characters where necessary, e.g., jiangbai, Sheng, Dan, Chou, tangqian |
| Analytical checking | Dialect-based humour, rhyme, comic timing, and local references checked against interview recordings, performance context, and field notes |
| Publication strategy | Short translated excerpts and paraphrases used in the article; sensitive or identifying details minimised |

# S9. Ethics and data-protection safeguards

| **Safeguard area** | **Statement** |
| --- | --- |
| Ethics approval | Human Research Ethics Committee, Universiti Pendidikan Sultan Idris, reference 2025-0661-01, approval period 28 February 2025 to 27 February 2026 |
| Pre-2025 materials | Treated as preliminary/contextual materials; included only where consent, verification, anonymisation, and ethical conditions permitted |
| Formal data | Formal doctoral fieldwork from March 2025 onward conducted within approval period |
| Anonymisation | P-codes, A-records, FN, VR, AR, and Photo codes used to preserve traceability without unnecessary identification |
| Excluded from supplement | Raw transcripts, audio/video files, full fieldnotes, unredacted photos, contact details, precise household addresses, and identifying non-public information |
| Data availability | Anonymised excerpts may be shared upon reasonable request where ethically permissible; raw data are not publicly released due to privacy and consent limitations |

# S10. Recommended supplementary-file statement for submission system

Suggested description to enter in the Frontiers supplementary upload field:

*Supplementary File 1 contains an anonymised methodological audit trail, including fieldwork overview, participant coding matrix, audience feedback record summary, coding tree, cross-material evidence matrix, visual evidence audit trail, translation procedure, and ethics/data-protection safeguards. No raw transcripts, identifiable images, audio/video files, contact details, or precise household locations are included.*

# S11. References cited in this supplementary file

This supplement is designed primarily as a methodological audit trail and does not introduce new substantive literature beyond the main manuscript. Any methodological sources mentioned in the main manuscript should be retained in the manuscript reference list and checked in Mendeley before submission.
